# Designed angiopoietin-1 variant, COMP-angiopoietin-1, rescues erectile function through healthy cavernous angiogenesis in a hypercholesterolemic mouse

**DOI:** 10.1038/srep09222

**Published:** 2015-03-18

**Authors:** Ji-Kan Ryu, Woo Jean Kim, Young Jun Koh, Shuguang Piao, Hai-Rong Jin, Sae-Won Lee, Min Ji Choi, Hwa-Yean Shin, Mi-Hye Kwon, Keehoon Jung, Gou Young Koh, Jun-Kyu Suh

**Affiliations:** 1 National Research Center for Sexual Medicine and Department of Urology, Inha University School of Medicine, Incheon 400-711, Republic of Korea; 2 Inha Research Institute for Medical Sciences, Inha University School of Medicine, Incheon 400-711, Republic of Korea; 3 National Research Laboratory of Vascular Biology and Stem Cells and Graduate School of Medical Science and Engineering, Korea Advanced Institute of Science and Technology (KAIST), Daejeon 305-701, Republic of Korea; 4 Department of Internal Medicine and Innovative Research Institute for Cell Therapy, Seoul National University Hospital, Seoul, Republic of Korea; 5 Biomedical Research Institute, Seoul National University Hospital, Seoul, Republic of Korea

## Abstract

Despite the advent of oral phosphodiesterase-5 inhibitors, curative treatment for erectile dysfunction (ED) remains unavailable. Recently, the link between ED and cardiovascular disease was unveiled and the main etiology of ED was found to be vasculogenic. Therefore, neovascularization is a promising strategy for curing ED. Angiopoietin-1 (Ang1) is an angiogenic growth factor that promotes the generation of stable and functional vasculature. Here, we demonstrate that local delivery of the soluble, stable, and potent Ang1 variant, COMP-Ang1 gene or protein, into the penises of hypercholesterolemic mice increases cavernous angiogenesis, eNOS phosphorylation, and cGMP expression, resulting in full recovery of erectile function and cavernous blood flow up to 8 weeks after treatment. COMP-Ang1-induced promotion of cavernous angiogenesis and erectile function was abolished in *Nos3^-/-^* mice and in the presence of the NOS inhibitor, L-NAME. COMP-Ang1 also restored the integrity of endothelial cell-cell junction by down-regulating the expression of histone deacetylase 2 in the penis of hypercholesterolemic mice and in primary cultured mouse cavernous endothelial cells. These findings constitute a new paradigm toward curative treatment of both cavernous angiopathy and ED.

The penis is a richly vascularized organ and erectile dysfunction (ED) is predominately a vascular disease^1^. Recently, a link between ED and cardiovascular disease was uncovered and both diseases were shown to share the same risk factors, including hypercholesterolemia, hypertension, diabetes mellitus, and smoking, with endothelial cell dysfunction being the common denominator between these two conditions^2^,^3^. These findings suggest that ED is another manifestation of systemic vascular disorder. In a prospective study of community-dwelling men 30 to 69 years of age^4^, hypercholesterolemia and age were strong independent predictors of ED at 25 years of follow up, and hypercholesterolemia was the most common risk factor in men with ED. It has been shown that hypercholesterolemia in men and animal models causes impairments in endothelium-dependent smooth muscle relaxation^5^, endothelial nitric oxide synthase (eNOS) enzyme activity^6^, and penile angiogenesis^7^,^8^, resulting in ED.

Although oral phosphodiesterase (PDE)-5 inhibitors, drugs that enhance the nitric oxide (NO)-cGMP pathway by inhibiting the hydrolysis of cGMP to inactive GMP, are generally effective and well-tolerated therapies for ED^9^,^10^,^11^, they are not cures for ED and have important limitations. Firstly, PDE5 inhibitors must be used on demand, thus hindering the spontaneity of the sexual act. Secondly, PDE5 inhibitors themselves do not augment NO formation; their effects rely on endogenous NO formation. Therefore, PDE5 inhibitors could fail to increase the level of cGMP above the necessary threshold if the bioavailability of endogenous NO is insufficient, which explains the failure of these drugs to relieve ED in men with severe cardiovascular disease, diabetes, or radical prostatectomy^12^,^13^. Finally, the use of PDE5 inhibitors is absolutely contraindicated in men who take nitrates, due to the possibility of extreme hypotension^14^.

Curative therapy for vasculogenic ED requires a new therapeutic strategy that reestablishes structural and functional microvasculature and augments endogenous NO bioactivity. However, patients with ED associated with hypercholesterolemia often have impaired endothelial function and reduced endothelium-derived NO release. Therefore, neovascularization has emerged as a strategy for treating vasculogenic ED and is anticipated to be more effective for patients with moderate to severe ED and to restore physiologic erections, i.e., spontaneity of the sexual act. Local intracavernous delivery of the vascular endothelial growth factor-A (VEGF-A) gene or protein has been shown to restore erectile function in animal models of vasculogenic ED^7^,^15^,^16^,^17^. However, treatment with exogenous VEGF-A often results in a pathologic angiogenesis producing leaky, inflamed, and disorganized blood vessels in experimental systems^18^,^19^, greatly compromising its therapeutic value. In comparison, angiopoietin-1 (Ang1), the ligand of the Tie2 receptor tyrosine kinase, is an angiogenic growth factor that specifically functions to generate a non-leaky, stable, and functional vasculature^19^,^20^,^21^,^22^,^23^. In addition, when administered with VEGF, Ang1 can counteract VEGF-induced side effects^23^,^24^, while having an additive effect on vessel formation^7^,^19^,^25^. However, our previous study revealed that a single intracavernous delivery of adenovirus-mediated Ang1 gene failed to induce an angiogenic response in the penis of a hypercholesterolemic rat^7^. Recently, we developed a soluble and potent Ang1 variant, cartilage oligomeric matrix protein (COMP)-Ang1^26^, which is more potent than native Ang1 in phosphorylating Tie2 in primary cultured endothelial cells. COMP-Ang1 was found to stimulate angiogenesis in the mouse corneal micropocket assay^26^ and to produce long-lasting, stable vascular enlargement associated with increased blood flow in the microvasculature of adult mice^27^. Upon COMP-Ang1 stimulation, Tie2 translocalization in endothelial cell-cell and cell-matrix contacts could be a main molecular event to induce the non-leaky, healthy angiogenesis and vascular enlargement^28^,^29^. Thus, COMP-Ang1 appears to be an effective alternative to native Ang1 for therapeutic applications *in vivo*.

In this study, we determined the effectiveness of COMP-Ang1 in promoting cavernous angiogenesis and restoring erectile function in a mouse model of hypercholesterolemic ED induced by a high-cholesterol diet. In addition, because Ang1-induced angiogenesis appears to require the generation of NO by activated eNOS^30^, we determined whether eNOS (encoded by *Nos3*) or NOS participated in COMP-Ang1-induced cavernous angiogenesis and subsequent restoration of erectile function using *Nos3^-/-^* mice fed a high-cholesterol diet or wild type hypercholesterolemic mice treated with NG-nitro-L-arginine methyl ester (L-NAME), a NOS inhibitor. Our results showed that a single injection of adenoviral COMP-Ang1 gene (ad-COMP-Ang1) or two successive injections of COMP-Ang1 recombinant protein into the corpus cavernosum induced complete and long-lasting recovery of erectile function and blood flow in hypercholesterolemic mice, which was accompanied by enhanced cavernous angiogenesis, eNOS phosphorylation, and cGMP expression. COMP-Ang1-induced restoration of erectile function and angiogenesis was dependent on eNOS or NOS. COMP-Ang1 also involved in the maintenance of integrity of endothelial cell-cell junction (EC junction) by down-regulating expression of histone deacelylase 2 (HDAC2) in the penis of hypercholesterolemic mice and in primary cultured mouse cavernous endothelial cells (MCECs) *in vitro*.

## Results

### Total cholesterol level

The serum total cholesterol concentrations of animals fed a diet containing 4% cholesterol with 1% cholic acid for 3 months were significantly higher than the concentrations in age-matched control mice fed a standard diet. All of the animals fed the high-cholesterol diet had similar total cholesterol levels regardless of the treatment given (see Supplemental Table 1 online).

### *In vivo* gene or protein expression in mouse corpora cavernosa

**Figure 1:**
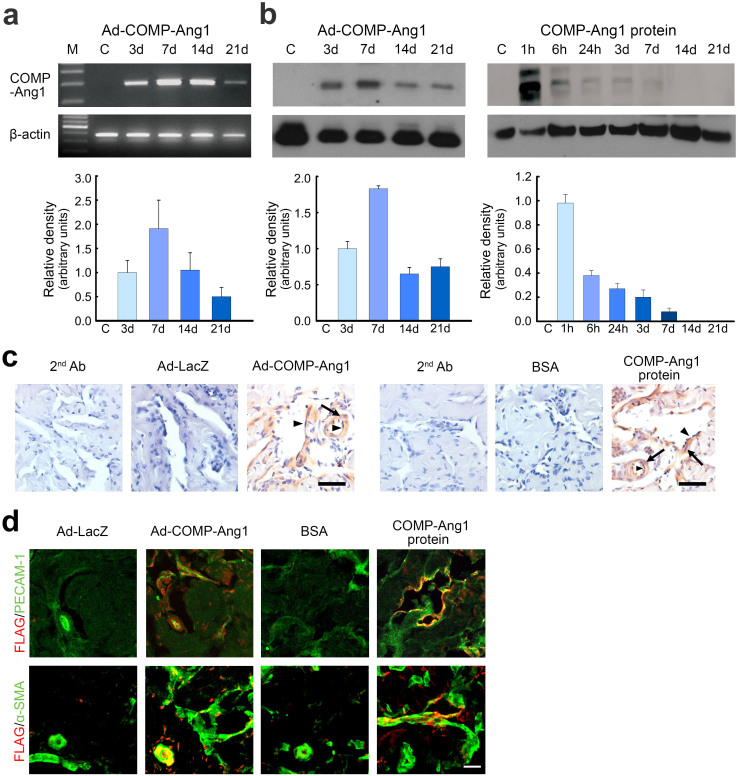
In vivo expression of COMP-Ang1 gene and protein. (a) Detection of COMP-Ang1-specific mRNA in the corpus cavernosum from hypercholesterolemic mice. PCR was performed with primers specific for COMP-Ang1. RNA was extracted from the corpus cavernosum 3, 7, 14, and 21 days after intracavernous administration of ad-COMP-Ang1 (2 × 10^8^ parts/20 μl). Densitometric data are presented as the relative ratio of COMP-Ang1 mRNA to β-actin mRNA. The relative ratio measured 3 days after injection of ad-COMP-Ang1 was arbitrarily set equivalent to 1. Bars represent the mean ± SEM of four independent experiments. M, marker. (b) Western blot analysis of the expression of COMP-Ang1 protein in cavernous tissues from hypercholesterolemic mice 1, 6, and 24 hours and 3, 7, 14, and 21 days after intracavernous injection of ad-COMP-Ang1 (left) or COMP-Ang1 protein (right). The relative ratio of COMP-Ang1 to β-actin measured 3 days after injection of ad-COMP-Ang1 or 1 hour after injection of COMP-Ang1 protein was arbitrarily set equivalent to 1. Bars represent the mean ± SEM of four independent experiments. (c) Anti-FLAG staining of cavernous tissue from hypercholesterolemic mice 7 days after intracavernous injection of ad-LacZ (2 × 10^8^ parts/20 μl) or ad-COMP-Ang1 (2 × 10^8^ parts/20 μl) and 1 hour after intracavernous injection of BSA (5.88 μg/20 μl) or COMP-Ang1 protein (5.88 μg/20 μl). 2^nd^ Ab, secondary antibody control; arrowheads, endothelial cells; arrows, smooth muscle cells. Scale bar = 50 μm. (d) Immunohistochemical staining of cavernous tissue using antibodies to FLAG (red), PECAM-1 (CD31; green), and α-actin (green) in each group. Scale bar = 50 μm.

Exogenous COMP-Ang1 mRNA or protein expression was detected in the corpus cavernosa of hypercholesterolemic mice 3, 7, 14, and 21 days after injection of ad-COMP-Ang1. The level of exogenous COMP-Ang1 transcript or protein expression was highest 7 days after injection and expression was still detectable at 21 days (Fig. 1a and Fig. 1b [left]).

We also evaluated the persistence of COMP-Ang1 protein in the corpus cavernosum at 1, 6, and 24 hours and 3, 7, 14, and 21 days after injection of COMP-Ang1 recombinant protein by western blot. The level of COMP-Ang1 protein peaked at the earliest time point assayed (1 hour) and was detectable up to 7 days after injection (Fig. 1b [right]).

To localize the COMP-Ang1 protein, either expressed from ad-COMP-Ang1 or injected as a recombinant protein, immunohistochemical staining for FLAG-tagged COMP-Ang1 was performed 7 days after intracavernous injection of ad-LacZ, or ad-COMP-Ang1, and 1 hour after intracavernous injection of BSA, or COMP-Ang1 protein. The FLAG-tagged protein was strongly expressed in endothelial cells and as well as in smooth muscle cells in both the ad-COMP-Ang1- and COMP-Ang1 protein-treated animals (Fig. 1c, d).

Cavernous Tie2 expression as assessed by immunohistochemical staining was significantly lower in hypercholesterolemic mice treated with ad-LacZ, BSA, or cholesterol diet only than in age-matched controls. Intracavernous injection of ad-COMP-Ang1 or COMP-Ang1 protein induced Tie2 expression in hypercholestrolemic mice (see Supplemental Fig. 1 online).

### COMP-Ang1 transfer completely restores erectile function

**Figure 2:**
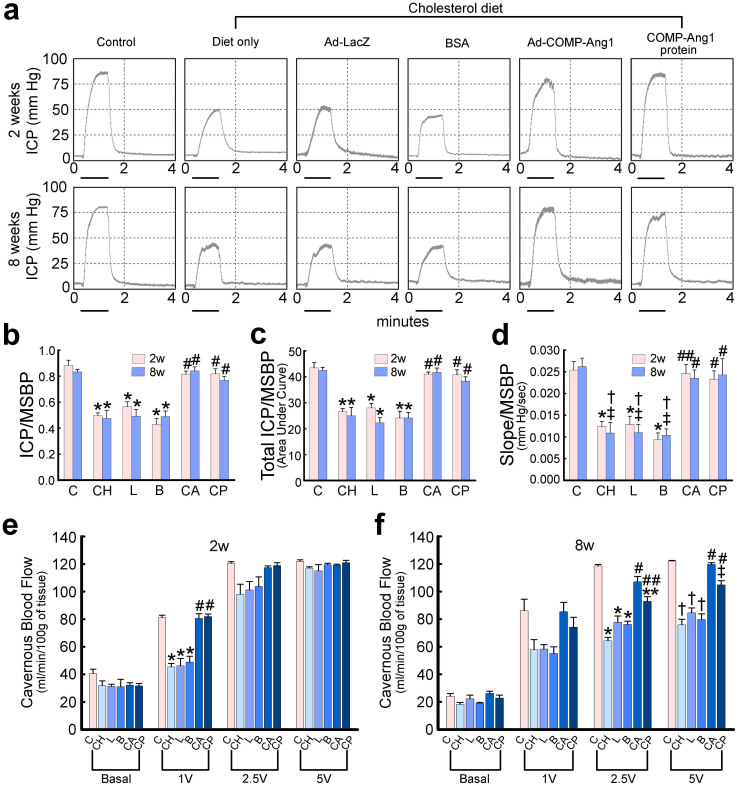
Adenoviral COMP-Ang1 gene or COMP-Ang1 protein transfer restores intracavernous pressure (ICP) and cavernous tissue blood flow elicited by electrical stimulation of the cavernous nerve. (a) Representative ICP responses for age-matched control (C) or hypercholesterolemic mice stimulated at 2 and 8 weeks after intracavernous injection of ad-LacZ (L, 2 × 10^8^ parts/20 μl), BSA (B, 5.88 μg/20 μl), ad-COMP-Ang1 (CA, 2 × 10^8^ parts/20 μl), COMP-Ang1 protein (CP, 5.88 μg/20 μl), or cholesterol diet only (CH). The stimulus interval is indicated by a solid bar. (b–d) Ratios of mean maximal ICP, total ICP (area under the curve), and slope to mean systolic blood pressure (MSBP) were calculated for each group. Each bar depicts the mean ± SEM for n = 8 animals per group. One-way ANOVA was used for statistical analysis. (b) Ratio of maximal ICP to MSBP. *p <0.01 vs C, CA, and CP groups, #p <0.01 vs CH, L, and B groups. (c) Ratio of total ICP (area under the curve) to MSBP. *p <0.01 vs C, CA, and CP groups, #p <0.01 vs CH, L, and B groups. (d) Ratio of slope to MSBP. *p <0.01 vs C and CA groups, †p <0.01 vs C group, ‡p <0.05 vs CA and CP groups, ##p <0.01 vs CH, L, and B groups, #P <0.05 vs CH, L, and B groups. (e, f) Laser-Doppler flowmetric analyses for cavernous tissue blood flow responses. Each bar depicts the mean ± SEM for n = 4 animals per group. One-way ANOVA was used for statistical analysis. (e) Two weeks after treatment. *p <0.01 vs C, CA, and CP groups, #p <0.01 vs CH, L, and B groups. (f) Eight weeks after treatment. *p <0.01 vs C and CA groups, #p <0.01 vs CH, L, and B groups, ##p <0.05 vs CH, L, and B groups, **p <0.01 vs C group, †p <0.01 vs C, CA, and CP groups, ‡p <0.05 vs C and CA groups.

In order to determine the physiological relevance of intracavernous injections COMP-Ang1 gene or protein, we evaluated erectile function during electrical stimulation of the cavernous nerve *in vivo*, 2 and 8 weeks after treatment. The optimal dose of ad-COMP-Ang1 or COMP-Ang1 protein needed to induce maximal erectile response was determined at 2 weeks post treatment. The highest response was recorded in hypercholesterolemic mice receiving a single injection of ad-COMP-Ang1 at a concentration of 2 × 10^8^ parts in 20 μl PBS or receiving repeated injections of COMP-Ang1 protein (days -3 and 0, 5.88 μg in 20 μl PBS; see Supplemental Fig. 2 online). A representative intracavernous tracing after stimulation of the cavernous nerve (5 V, 12 Hz, 1 ms) for 1 min in age-matched control or hypercholesterolemic mice 2 and 8 weeks after treatment is shown in Fig. 2a. The ratios of maximal intracavernous pressure, total intracavernous pressure, and slope to mean systolic blood pressure were significantly lower in hypercholesterolemic mice treated with ad-LacZ, BSA, or cholesterol diet only than in age-matched controls. Ad-COMP-Ang1 or COMP-Ang1 protein injection completely normalized all erection parameters up to 8 weeks after treatment (Fig. 2b–d). No differences in systemic blood pressure were detected among the six experimental groups (see Supplemental Table 1 online).

We also evaluated the effect of cavernous nerve stimulation on cavernous blood flow *in vivo* 2 and 8 weeks after treatment. During electrical stimulation of the cavernous nerve (1–5 V, 12 Hz, 1 ms), cavernous tissue blood flow was significantly lower in hypercholesterolemic mice treated with ad-LacZ, BSA, or cholesterol diet only than in age-matched controls. Intracavernous administration of ad-COMP-Ang1 completely normalized cavernous tissue blood flow up to 8 weeks after treatment, whereas hypercholesterolemic mice treated with COMP-Ang1 protein showed complete recovery of cavernous blood flow at 2 weeks and partial recovery at 8 weeks (Fig. 2e, f).

### COMP-Ang1 transfer restores cavernous endothelial content and EC junction proteins

**Figure 3:**
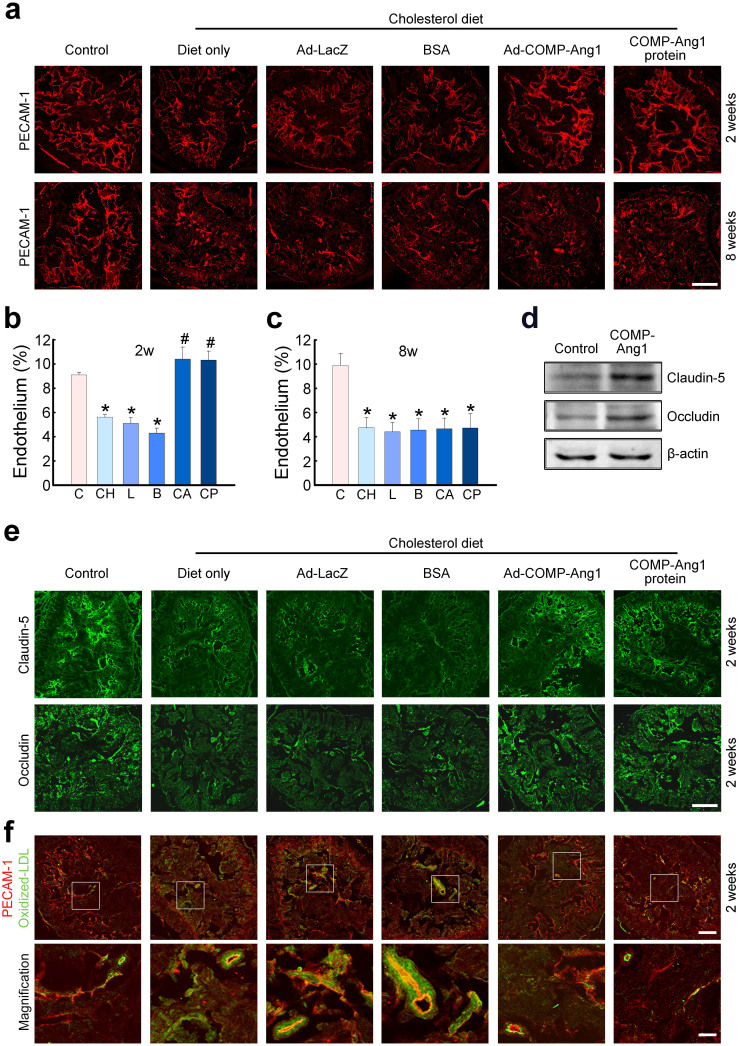
Adenoviral COMP-Ang1 gene or COMP-Ang1 protein transfer increases cavernous endothelial content. (a) Anti-PECAM-1 staining of cavernous tissue from age-matched control (C) or hypercholesterolemic mice 2 and 8 weeks after intracavernous injection of ad-LacZ (L, 2 × 10^8^ parts/20 μl), BSA (B, 5.88 μg/20 μl), ad-COMP-Ang1 (CA, 2 × 10^8^ parts/20 μl), COMP-Ang1 protein (CP, 5.88 μg/20 μl), or cholesterol diet only (CH). Scale bar = 200 μm. (b, c) Quantitative analysis of endothelial cell content in cavernous tissue was performed using an image analyzer. Each bar depicts the mean ± SEM for n = 8 animals per group. One-way ANOVA was used for statistical analysis. (b) Two weeks after treatment. *p <0.01 vs C, CA, and CP groups, #p <0.01 vs CH, L, and B groups. (c) Eight weeks after treatment. *p <0.01 vs C group. (d) COMP-Ang1 protein increases expression of claudin-5 and occludin in MCECs. Representative Western blot for occludin and claudin-5. The cells were incubated with COMP-Ang1 protein (400 ng/ml/d) for three consecutive days and cells were harvested 24 hours after treatment. Results were similar from four independent experiments. (e) Immunohistochemical staining of cavernous tissue using antibodies to claudin-5 (green) and occludin (green) in each group. Scale bar = 200 μm. (f) Immunohistochemical staining of cavernous tissue using antibodies to oxidized-LDL (green) and PECAM-1 (CD31; red) in each group. Scale bar = 200 μm (upper column) or 50 μm (lower column).

Immunohistochemical staining of cavernous tissue with an antibody to platelet/endothelial cell adhesion molecule (PECAM)-1 was performed in age-matched control and hypercholesterolemic mice, 2 and 8 weeks after treatment. We found significantly fewer endothelial cells in the hypercholesterolemic mice treated with ad-LacZ, BSA, or cholesterol diet only than in age-matched control mice. Intracavernous administration of ad-COMP-Ang1 or COMP-Ang1 protein completely restored cavernous endothelial content 2 weeks after treatment (Fig. 3a, b). However, 8 weeks after administration of either ad-COMP-Ang1 or COMP-Ang1 protein, cavernous endothelial content returned to baseline levels (Fig. 3a, c).

We recently have found that derangements in EC junctions are important pathophysiologic mechanisms involved in ED from vascular causes, such as hypercholesterolemia and diabetes^8^,^31^. In the present study, we determined the role of COMP-Ang1 on the expression of tight junction proteins in primary cultured MCECs *in vitro*. Treatment of MCECs with COMP-Ang1 protein (400 ng/ml/d for 3 days) induced increase in the protein expression of claudin-5 and occludin (Fig. 3d). Similar to this finding, intracavernous injection of ad-COMP-Ang1 or COMP-Ang1 protein induced recovery of claudin-5 and occludin expression in the penis of hypercholesterolemic mice 2 weeks after treatment (Figure 3e). We performed immunofluorescent double staining with antibodies to oxidized-LDL and PECAM-1 to examine the effect of COMP-Ang1 on cavernous endothelial permeability. We observed a significant increase in the extravasation of oxidized-LDL in the corpus cavernosum tissue of hypercholesterolemic mice treated with ad-LacZ, BSA, or cholesterol diet only than in age-matched controls. Ad-COMP-Ang1 or COMP-Ang1 protein injection decreased cavernous endothelial permeability to oxidized LDL in hypercholestrolemic mice (Figure 3f).

### COMP-Ang1 transfer increases the number of dividing endothelial cells

To evaluate whether the COMP-Ang1-induced increase in cavernous endothelial content resulted from endothelial cell proliferation, we used immunostaining to determine the number of endothelial cells positive for phosphohistone H3 (a nuclear protein indicative of cell proliferation). Numerous phosphohistone H3-positive endothelial cells were detected in the cavernous sinusoids of hypercholesterolemic mice 2 weeks after intracavernous injection of ad-COMP-Ang1 (see Supplemental Fig. 3a, c online). In contrast, a few or virtually no phosphohistone H3-positive endothelial cells were noted in age-matched control and hypercholesterolemic mice treated with ad-LacZ, BSA, COMP-Ang1 protein, or cholesterol diet only (see Supplemental Fig. 3a, c online). We further investigated whether COMP-Ang1 protein could induce endothelial cell proliferation at earlier time points and found a significant increase in phosphohistone H3-positive endothelial cells as well as an increase in PECAM-1 expression 3 and 6 hours after injection of COMP-Ang1 protein (see Supplemental Fig. 3b, d online). These findings indicate that the COMP-Ang1-induced increase in cavernous endothelial content resulted from endothelial cell proliferation.

### COMP-Ang1 transfer induces eNOS phosphorylation and increases cGMP concentration

**Figure 4:**
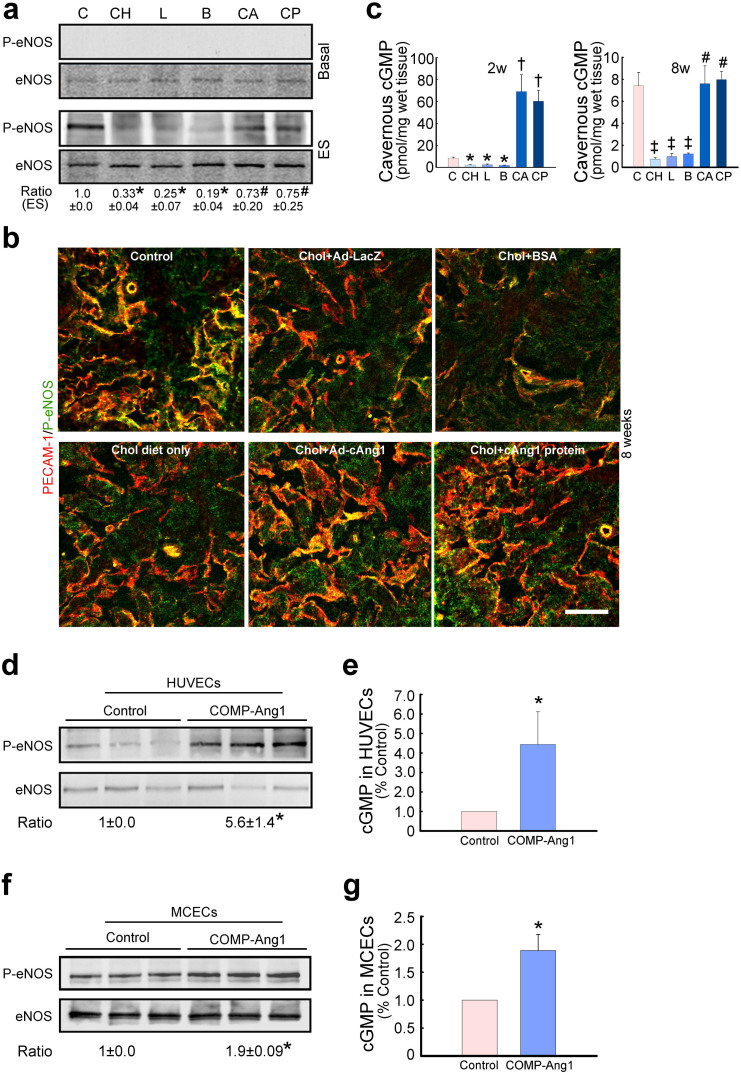
Adenoviral COMP-Ang1 gene or COMP-Ang1 protein transfer induces eNOS phosphorylation and increases cGMP concentration. (a–c) eNOS phosphorylation and cGMP expression in corpus cavernosum tissue. (a) Western blot analysis demonstrating the relative abundance of phospho-eNOS (p-eNOS, Ser1177) in age-matched control (C) or hypercholesterolemic mice 7 days after intracavernous injection of ad-LacZ (L, 2 × 10^8^ parts/20 μl), BSA (B, 5.88 μg/20 μl), ad-COMP-Ang1 (CA, 2 × 10^8^ parts/20 μl), COMP-Ang1 protein (CP, 5.88 μg/20 μl), or cholesterol diet only (CH). Data are representative of four independent experiments. The ratios indicate the relative ratio of p-eNOS to eNOS as determined by densitometry. The relative ratio of the control group (C) was arbitrarily set equivalent to 1. Kruskal-Wallis test was used for statistical analysis. *p <0.01 vs C, CA, and CP groups, #p <0.01 vs CH, L, and B groups. (b) Immunohistochemical staining of cavernous tissue using antibodies to PECAM-1 (CD31; red) and p-eNOS (green) in age-matched control or hypercholesterolemic mice 8 weeks after intracavernous injection of each gene or protein. Results were similar for three independent experiments. Scale bar = 100 μm. (c) Cavernous cGMP concentrations. Each bar depicts the mean ± SEM for n = 4 animals per group. One-way ANOVA was used for statistical analysis. *p <0.01 vs C, CA, and CP groups, †p <0.01 vs C group, ‡p <0.01 vs C, CA, and CP groups, #p <0.01 vs CH, L, and B groups. (d–g) eNOS phosphorylation and cGMP expression in HUVECs and MCECs. The cells were incubated with COMP-Ang1 protein (400 ng/ml/d) for three consecutive days and cells were harvested 24 hours after treatment. (d, f) Western blot analysis for p-eNOS and eNOS. Data are the mean ± SEM of three independent experiments. Mann-Whitney *U*-test was used for statistical analysis. *p <0.01 vs control group. (e, g) cGMP concentrations in HUVECs and MCECs. Each bar represents the mean ± SEM for n = 6 per group. Mann-Whitney *U*-test was used for statistical analysis. *p <0.05 vs control group.

We evaluated the expression of phospho-eNOS and eNOS in the corpus cavernosum of age-matched control and hypercholesterolemic mice by immunoblot analysis 2 weeks after treatment. Almost no phospho-eNOS expression was detected in the corpus cavernosum tissue of all experimental groups at baseline (Fig. 4a). In hypercholesterolemic mice treated with ad-LacZ, BSA, or cholesterol diet only, the levels of phospho-eNOS (Ser1177) in the cavernous tissues harvested immediately after electrical stimulation of the cavernous nerve were significantly lower than in the age-matched controls, as determined by the ratio of phospho-eNOS to total eNOS.

Intracavernous administration of ad-COMP-Ang1 or COMP-Ang1 protein significantly increased endogenous eNOS phosphorylation as compared to hypercholesterolemic mice treated with ad-LacZ, BSA, or cholesterol diet only, although not to the level found in the age-matched controls (Fig. 4a). No significant differences were found in basal or stimulated total eNOS protein expression among the six experimental groups (Fig. 4a). Similar to the results of immunoblot analysis, immunohistochemical staining of the corpus cavernosum tissues harvested immediately after electrical stimulation of the cavernous nerve revealed an increase in cavernous phospho-eNOS expression in hypercholesterolemic mice 8 weeks after treatment with either ad-COMP-Ang1 or COMP-Ang1 protein (Fig. 4b).

Cavernous cGMP decreased significantly in hypercholesterolemic mice treated with ad-LacZ, BSA, or cholesterol diet only compared with that in the age-matched controls. At 2 weeks post intracavernous injection of ad-COMP-Ang1 or COMP-Ang1 protein, cGMP concentrations increased dramatically by approximately 8.3- or 7.3-fold, respectively, as compared with controls (Fig. 4c). At 8 weeks post injection, cGMP concentration in hypercholesterolemic mice treated with either ad-COMP-Ang1 or COMP-Ang1 protein returned to the control level, but was still significantly higher than that in mice treated with ad-LacZ, BSA, or cholesterol diet only (Fig. 4c).

We also determined the effects of COMP-Ang1 protein on eNOS phosphorylation and cGMP concentration in human umbilical vein endothelial cells (HUVECs) and MCECs. The cells treated with COMP-Ang1 protein (400 ng/ml/d for 3 days) showed a significant increase in eNOS phosphorylation and cGMP concentration by approximately 5.6- and 4.4-fold in HUVECs, and 1.9- and 1.8-fold in MCECs, respectively, as compared to the cells treated with control buffer alone (Fig. 4d–g). In agreement with our *in vivo* results, total eNOS expression in HUVECs and MCECs was unchanged after treatment with COMP-Ang1 protein (Fig. 4d, f).

### COMP-Ang1 gene-induced cavernous angiogenesis and recovery of erectile function is eNOS- or NOS-dependent

**Figure 5:**
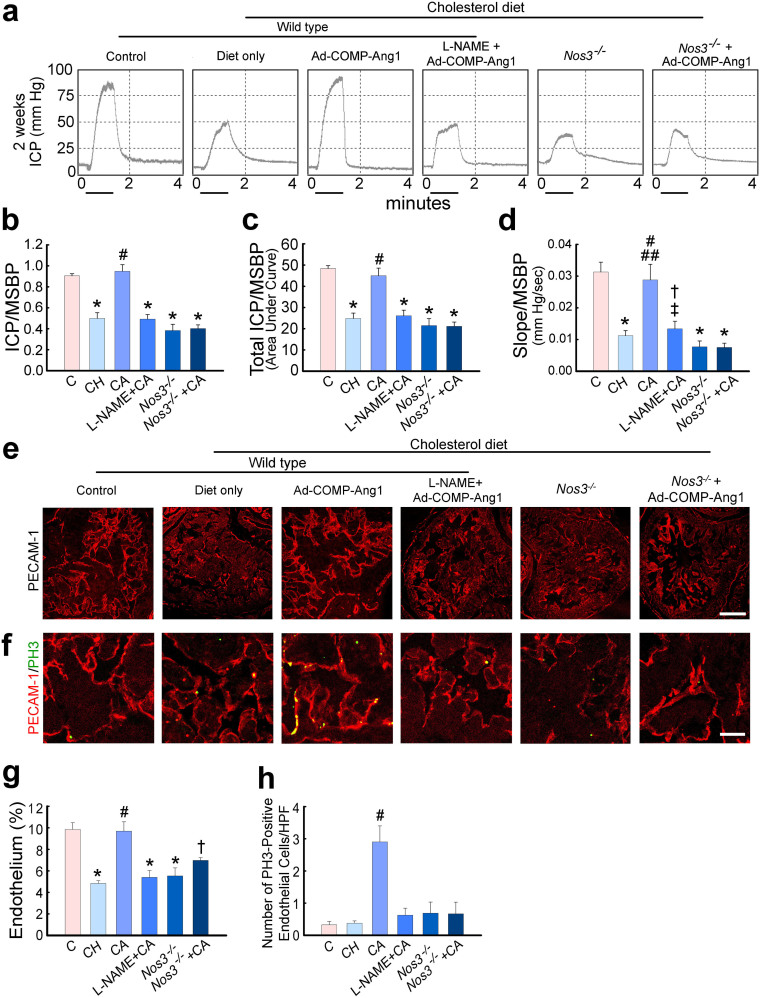
Adenoviral COMP-Ang1 gene-induced cavernous angiogenesis and recovery of erectile function is eNOS- or NOS-dependent. (a) Representative intracavernous pressure (ICP) responses elicited by electrical stimulation of the cavernous nerve for age-matched controls (C), hypercholesterolemic mice receiving intracavernous injection of ad-COMP-Ang1 (CA) or cholesterol diet only (CH), L-NAME-treated hypercholesterolemic mice receiving ad-COMP-Ang1 (L-NAME + CA), Nos3^-/-^ mice fed a cholesterol diet (Nos3^-/-^), or Nos3^-/-^ mice fed a high cholesterol diet and treated with ad-COMP-Ang1 (Nos3^-/-^ + CA). One-way ANOVA was used for statistical analysis. (b–d) The ratios of mean maximal ICP, total ICP (area under the curve), and slope to mean systolic blood pressure (MSBP) were calculated for each group. Each bar depicts the mean ± SEM for n = 4 animals per group. (b) Ratio of maximal ICP to MSBP. *p <0.01 vs C and CA groups, #p <0.01 vs CH, L-NAME + CA, Nos3^-/-^, and Nos3^-/-^ + CA groups. (c) Ratio of total ICP (area under the curve) to MSBP. *p <0.01 vs C and CA groups, #p <0.01 vs CH, L-NAME + CA, Nos3^-/-^, and Nos3^-/-^ + CA groups. (d) Ratio of slope to MSBP. *p <0.01 vs C and CA groups, †p <0.01 vs C group, ‡p <0.05 vs CA group, #p <0.01 vs CH, Nos3^-/-^, and Nos3^-/-^ + CA groups, ##p <0.05 vs L-NAME + CA group. (e, f) Immunohistochemical staining of cavernous tissue using antibodies to PECAM-1 (CD31; red) and phosphohistone H3 (PH3; green) in each group. Scale bar = 200 μm for (e) and 50 μm for (f). (g) Quantitative analysis of endothelial cell content. Each bar depicts the mean ± SEM for n = 4 animals per group. *p <0.01 vs C and CA groups, †p <0.05 vs CA group, #p <0.01 vs CH, L-NAME + CA, and Nos3^-/-^ groups. (h) Number of PH3-immunopositive endothelial cells per high-power field (HPF, screen magnification ×400). Each bar depicts the mean ± SEM for n = 4 animals per group. #p <0.01 vs C, CH, L-NAME + CA, Nos3^-/-^, and Nos3^-/-^ + CA groups.

We determined whether eNOS or NOS participated in COMP-Ang1-induced cavernous angiogenesis and subsequent restoration of the erectile response in *Nos3^-/-^* mice fed a high-cholesterol diet or L-NAME-treated wild type C57BL/6J mice fed a high-cholesterol diet. Physiologic erection studies indicated that *Nos3^-/-^* or L-NAME-treated wild type mice transfected with ad-COMP-Ang1 failed to recover erectile function following treatment with ad-COMP-Ang1 (Fig. 5a–d). Moreover, ad-COMP-Ang1-induced enhancement of cavernous angiogenesis was remarkably diminished in *Nos3^-/-^* mice fed a high-cholesterol diet or abolished in L-NAME-treated wild type mice fed a high-cholesterol diet, as evidenced by immunohistochemical staining for PECAM-1 and phosphohistone H3 (Fig. 5e–h). These findings suggest that the recovery of erectile function promoted by COMP-Ang1-induced cavernous angiogenesis depends on eNOS or NOS.

### COMP-Ang1 transfer increases EC junction proteins through down-regulation of HDAC2

HDACs are known to involve in the regulation of junctional integrity^32^,^33^. The cavernous expression of HDAC2 was significantly higher in hypercholesterolemic mice treated with ad-LacZ or cholesterol diet only than in age-matched controls. Intracavernous administration of ad-COMP-Ang1 significantly decreased HDAC2 expression in the corpus cavernosum tissue of hypercholesterolemic mice (see Supplemental Fig. 4a online). We determined which cell type is a main source of HDAC2 expression in hypercholesterolemic penis. Immunofluorescent double staining of cavernous tissue with antibodies to HDAC2 and PECAM-1 showed that a significant proportion of the HDAC2 expression overlapped with endothelial cells of the cavernous sinusoids and cavernous artery, but rarely overlapped with smooth muscle cells (see Supplemental Fig. 4b, c online).

**Figure 6:**
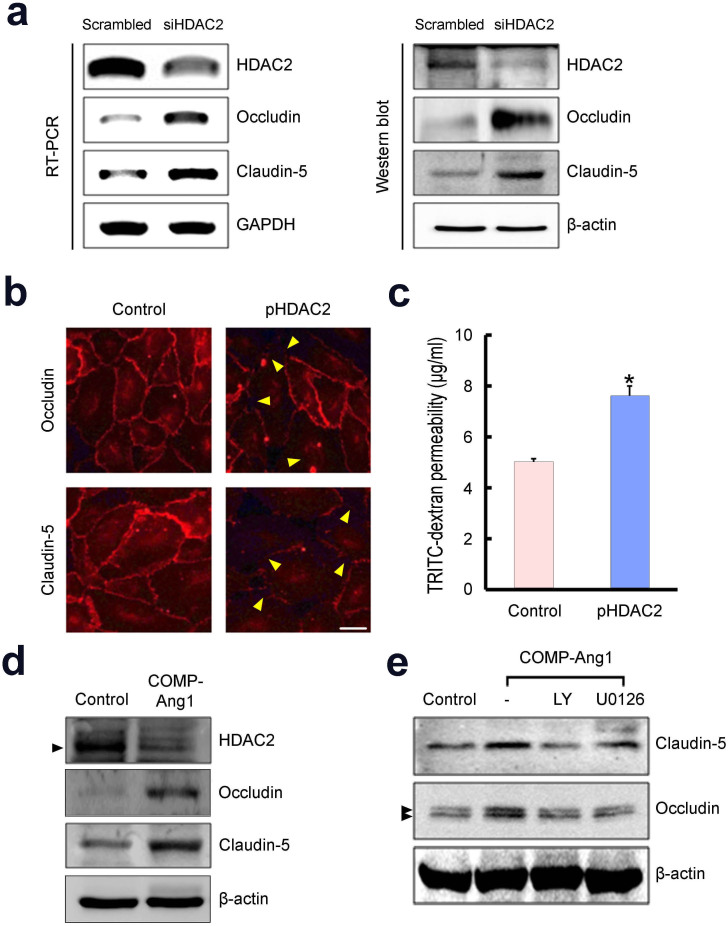
HDAC2 regulates the expression of tight junction proteins in MCECs. (a) siRNA-mediated silencing of HDAC2 increases the expression of tight junctions in primary cultured MCECs. (Left) Representative gel picture showing the gene expression of occludin and claudin-5. GAPDH was used as an internal control for RT-PCR. (Right) Representative Western blot for occludin and claudin-5. β-actin was used as an internal control for Western blot. After serum starvation for 24 hours, MCECs were transfected with scrambled siRNA or siRNA specific to HDAC2 for 48 hours. Results were similar from four independent experiments. (b) Overexpression of HDAC2 with plasmid encoding HDAC2 (pHDAC2) decreases the expression of tight junction proteins in MCECs. Immuocytochemical staining of MCECs performed with antibodies to occludin and claudin-5. Arrow heads denote disorganized tight junctions. Results were similar from four independent experiments. Scale bar = 50 μm. (c) Overexpression of HDAC2 with pHDAC2 increases vascular endothelial permeability in MCECs. Vascular endothelial permeability was determined by using the passage of rhodamine B isothiocyanate (RITC)-labeled dextran through monolayer of MCECs. Each bar depicts the mean ± SEM of four independent experiments. Mann-Whitney *U*-test was used for statistical analysis. *P <0.01 vs control group. (d) COMP-Ang1 protein (400 ng/ml) decreases HDAC2 expression and increases expression of occludin and claudin-5 in MCECs. Representative Western blot for HDAC2, occludin, and claudin-5. Results were similar from four independent experiments. (e) COMP-Ang1 protein-mediated increase in tight junction expression is dependent on PI3K/Akt or MEK/ERK pathway. MCECs were pretreated with chemical inhibitor of PI3K/Akt pathway (LY 294002) or MEK/ERK pathway (U0126), and then treated with COMP-Ang1 protein (400 ng/ml). Results were similar from four independent experiments. MCECs, mouse cavernous endothelial cells.

Next, we determined the role of HDAC2 on the expression of tight junction proteins in primary cultured MCECs *in vitro*. Both RT-PCR and Western blot analysis revealed that the expression of occludin and claudin-5 was significantly higher in MCECs transfected with HDAC2 siRNA than in cells transfected with scrambled siRNA (Fig. 6a). To examine the effect of HDAC2 overexpression on the integrity of EC junctions, we transfected HDAC2 plasmid into the primary cultured MCECs. In contrast to the findings from control group showing well-organized and evenly distributed EC junctions between cell borders, HDAC2 overexpression group revealed a weak and disorganized distribution of tight junction protein in MCECs (Fig. 6b). Then, we measured vascular endothelial permeability by using the passage of rhodamine B isothiocyanate-dextran through monolayer of MCECs and found that HDAC2 overexpression significantly increased vascular endothelial permeability (Fig. 6c). Similar to the results from *in vivo* study in hypercholesterolemic mice, treatment of MCECs with COMP-Ang1 protein decreased HDAC2 expression, which resulted in increase in the protein expression of occludin and claudin-5 (Fig. 6d). COMP-Ang1-induced increase in occludin and claudin-5 expression in MCECs was abolished by blockade of PI3K/Akt pathway with the chemical inhibitor LY294002. Pharmacologic inhibition of MEK/ERK signaling by U0126 also completely blocked COMP-Ang1-mediated restoration of occludin, and partially blocked cluadin-5, in MCECs (Fig. 6e). Knockdown of HDAC2 with siRNA did not affect on cell viability, migration, and tube formation in MCECs (see Supplemental Fig. 5 online).

### Biodistribution of adenovirus-delivered COMP-Ang1 DNA

We compressed the penis at the base with a vascular clamp immediately before injection and the clamp remained in place for 30 minutes to restrict blood flow out of the penis. Then, we evaluated the biodistribution of ad-COMP-Ang1 after intracavernous injection in a separate group of hypercholesterolemic mice and found that ad-COMP-Ang1 mRNA was expressed in a variety of major organs 7 or 14 days, but not 21 days, after injection (see Supplemental Fig. 6 online).

## Discussion

We successfully restored corpus cavernosum tissue both structurally and functionally in a mouse model of hypercholesterolemic ED using local delivery of an angiogenic factor gene or protein. The structural improvement (restoration of endothelial cell arrangement through enhanced endothelial cell proliferation) was noted for up to 2 weeks and stabilized 8 weeks after treatment with COMP-Ang1, whereas the functional or physiological improvements (restoration of erectile function via an increase in phospho-eNOS expression, cGMP level, and cavernous blood flow) persisted for up to 8 weeks post treatment.

In order to determine whether the improved erectile response was related to an increase in the number of endothelial cells, we assessed the expression of PECAM-1 by immunohistochemical analysis. We found a significantly smaller area of endothelial cells in hypercholesterolemic mice than in age-matched control mice. Reported mechanisms for impaired angiogenesis associated with hypercholesterolemia are endothelial dysfunction and decreased endothelium-derived NO formation^34^; elevation of asymmetric dimethylarginine^35^, an endogenous inhibitor of NOS; degradation of NO and deterioration of endothelial proliferation by superoxide anion production^36^,^37^; and inhibition of endothelial cell migration mediated by oxidized low-density lipoprotein and lysophosphatidylcholine^38^,^39^. The influence of ad-COMP-Ang1 or COMP-Ang1 protein on angiogenesis was confirmed by the complete recovery of PECAM-1-positive endothelial density in the corpus cavernosum. Our immunohistological examination of phosphohistone H3 expression revealed that the COMP-Ang1-induced increase in cavernous endothelial content was the result of endothelial proliferation, which is consistent with our recent report^27^.

It was reported that endothelium-derived NO is a crucial mediator of angiogenesis and *Nos3^-/-^* mice have shown to exhibit impairment in postnatal angiogenesis in response to growth factors^40^. In the present study, COMP-Ang1 failed to induce cavernous angiogenesis and restore erectile function in *Nos3^-/-^* mice or L-NAME-treated wild type mice fed a high-cholesterol diet, suggesting that recovery of erectile function mediated by COMP-Ang1-induced cavernous angiogenesis depends on eNOS or NOS. This finding is similar to results of a previous study showing that Ang1-induced angiogenesis was abolished or reduced in the presence of the NOS inhibitor L-NAME or in *Nos3^-/-^* mice in subcutaneous Matrigel implants *in vivo*^30^. The NO-cGMP system is the principal mediator of penile erection^41^. We and other investigators have reported that angiogenic factors such as Ang1 or VEGF-A^7^, and the dominant-negative RhoA mutant^42^, enhance penile erection in the hypercholesterolemic or diabetic rat through a NO-cGMP-dependent mechanism. In the present study, intracavernous transfer of the COMP-Ang1 gene or protein significantly increased cavernous nerve stimulation-induced endogenous eNOS phosphorylation in the hypercholesterolemic mouse penis, whereas no changes were noted in the total amount of eNOS, regardless of treatment. This finding agrees with previous reports by us and other investigators^7^,^43^, documenting a change in phospho-eNOS but not total eNOS protein expression after intracavernous injection of either VEGF-A or Ang1 in the penis of hypercholesterolemic rats or castrated mice. In this study, intracavernous administration of ad-COMP-Ang1 or COMP-Ang1 protein also dramatically increased the concentration of cGMP. This increased formation of cGMP, the crucial step in the initiation of NO-mediated smooth muscle relaxation, is clear evidence of excellent erectile response to cavernous nerve stimulation in the hypercholesterolemic mice. Similarly, COMP-Ang1 protein also induced a significant increase in eNOS phosphorylation and cGMP concentration in HUVECs and MCECs *in vitro*.

Ang1 is known to reduce endothelial permeability and promote blood vessel stability^28^,^29^. We recently reported in diabetic mice that intracavernous injection of COMP-Ang1 protein significantly decreased cavernous endothelial permeability by restoring EC junction proteins^31^. Similar to this finding, COMP-Ang1 profoundly decreased extravasation of oxidized LDL in the penis of hypercholesterolemic mice. However, the molecular mechanisms for how Ang1 regulates EC junctions are not fully delineated. Several lines of evidences suggest that HDACs are involved in the regulation of junctional integrity^32^,^33^. Valproic acid, a HDAC inhibitor, is known to attenuate blood-brain barrier disruption in a rat model of cerebral ischemia through inhibition of tight junction degradation^32^. HDAC inhibitor sodium butyrate is also reported to up-regulate the expression of tight junction proteins in fibroblasts^33^. However, little is known in regard to the specific role of HDAC subtypes in the regulation of EC junctions. During the identification of interaction between HDAC subtypes and Ang1, we found that the expression of HDAC2 was significantly higher in the penis of hypercholesterolemic mice than in controls, which was returned to the baseline level after treatment with COMP-Ang1. Overexpression of HDAC2 induced disruption of EC junctions and increased vascular endothelial permeability in MCECs. Treatment of MCECs with COMP-Ang1 significantly decreased HDAC2 expression and increased the expression of occludin and claudin-5. These results suggest that COMP-Ang1 has an effect to restore healthy EC junctions in the penis through inhibition of HDAC2.

Ang1 is known to enhance survival of endothelial cells and to reduce permeability of these cells through activation of PI3K/Akt pathway, whereas activation of the ERK pathway is involved in Ang1-mediated angiogenesis by inducing endothelial cell sprouting^44^. Similar to these findings, the barrier protective effect of COMP-Ang1 via induction of occludin and claudin-5 was abolished by inhibition of Akt pathway in primary cultured MCECs. We also found that pharmacologic inhibition of ERK pathway partially diminished Ang1-mediated restoration of EC junction protein, which indicates that activation of ERK pathway is also involved in Ang1-mediated anti-permeability function.

For clinical applications, it is important to determine whether the angiogenic factor should be given as a gene or a protein preparation. In the assay evaluating the biodistribution of viral vectors, intracavernous administration of ad-COMP-Ang1 resulted in mRNA expression in various major organs 7 and 14 days after injection, raising concerns about the utility of adenoviral vector-mediated therapy for non-life threatening disease, such as ED. Thus, the complete and long-lasting restoration of erectile function achieved after two successive intracavernous injections of COMP-Ang1 protein is intriguing; from a clinical standpoint, the use of a therapeutic protein for treating human ED could promote safer and more accurate therapeutic angiogenesis than that produced by gene therapy.

In conclusion, intracavernous delivery of adenoviral COMP-Ang1 gene or COMP-Ang1 protein increased Ang1 mRNA and/or protein expression, the ratio of phospho-eNOS to eNOS, cGMP expression, as well as healthy cavernous angiogenesis, resulting in physiologically relevant changes in erectile function and cavernous tissue blood flow as evidenced by electrical stimulation of cavernous nerve up to 8 weeks after treatment. These findings support the concept of angiogenic factor therapy as curative therapy for ED. Local therapy with COMP-Ang1 is highly promising as a definitive treatment modality for vascular disease-induced ED.

## Methods

Methods details are given in the Supplemental information online.

### Generation of COMP-Ang1 adenovirus and COMP-Ang1 recombinant protein

Recombinant adenovirus expressing FLAG-tagged COMP-Ang1 or bacterial β-gal was constructed, and COMP-Ang1 recombinant protein was prepared, as previously described^27^.

### Cell culture experiments

HUVECs or MCECs were prepared and maintained as previously described^45^,^46^. *In vitro* angiogenesis, cell proliferation, and cell permeability assay were performed as described in the Supplemental information online.

### Animals and treatments

Specific pathogen-free C57BL/6J and *Nos3^-/-^* (C57BL/6J genetic background) mice were purchased from Orient Bio (Gyeonggi, South Korea) and from The Jackson Laboratory (Bar Harbor, ME, USA) and bred in our pathogen-free animal facility. Animal care and all experimental procedures were conducted in accordance with the approval and guidelines of the INHA Institutional Animal Care and Use Committee (INHA IACUC) of the Medical School of Inha University. Control animals were fed a normal diet and experimental animals were fed a diet containing 4% cholesterol and 1% cholic acid (Feed Lab. Co., Gyeonggi, South Korea) for 3 months. Treatments of mice were performed as described in the Supplemental information online.

### Physiologic erection and inhibition studies

The mice from each cholesterol group and their age-matched controls were anesthetized and systemic blood pressure was measured using a noninvasive tail-cuff system (Visitech Systems, Apex, NC, USA). The validity of this system has been demonstrated previously^47^. Erectile function parameters, such as the ratios of maximal ICP, total ICP, and slope to MSBP, were measured by cavernous nerve electrical stimulation as previously described^7^,^48^. For inhibition studies, a separate group of hypercholesterolemic mice was given L-NAME (0.1 mg/ml) in drinking water for 11 days beginning 1 h after intracavernous injection of ad-COMP-Ang1. L-NAME was withdrawn 3 days prior to the measurement of erectile function; this time period has been shown to reduce the L-NAME concentrations in the blood by 80–90%^49^. We also evaluated erectile function in *Nos3^-/-^* mice fed a high cholesterol diet 2 weeks after intracavernous injection of ad-COMP-Ang1.

### Measurement of cavernous blood flow

Cavernous blood flow was measured by laser Doppler flow probe (1.2-mm diameter, Type N; Transonic Systems Inc. Chicago, IL, USA) after cavernous nerve stimulation (0–5 V, 12 Hz, 1 ms). Three electrostimulations were replicated at intervals of 10 min, and the mean tissue perfusion rate (ml/min per 100 g of tissue) during cavernous nerve stimulation was calculated.

### Immunohistochemistry, RT-PCR, Western Blot, and cGMP determination

Immunohistochemistry, RT-PCR, Western blot, and cGMP determination were performed as described in the Supplemental information online.

### Statistical analysis

Results are expressed as mean ± SEM. For parametric data, inter-group comparisons were made by one-way ANOVA followed by Newman-Keuls post-hoc tests. We used the Mann-Whitney *U*-test or Kruskal-Wallis test to compare nonparametric data. Probability values less than 5% were considered significant. We performed statistical analyses using SigmaStat 3.5 software (Systat Software Inc., Richmond, CA, USA).

## Author Contributions

J.-K.R., W.J.K., G.Y.K. and J.-K.S. designed research; J.-K.R., W.J.K., S.P., H.-R.J., M.J.C., H.-Y.S. and M.-H.K. performed research; Y.J.K. and K.J. contributed vital new reagents; J.-K.R., W.J.K. and S.-W.L. collected and interpreted data; J.-K.R., W.J.K., G.Y.K. and J.-K.S. wrote the manuscript.

## Supplementary Material

Supplementary Information Supplemental methods, table, and figures
