# Supplementary material for: Designed angiopoietin-1 variant, COMP-angiopoietin-1, rescues erectile function through healthy cavernous angiogenesis in a hypercholesterolemic mouse
Source: Sci Rep. 2015 Mar 18;5:9222. doi: 10.1038/srep09222 (PMC4363832; doi:10.1038/srep09222)
Supplement: Supplementary Information — Supplemental methods, table, and figures [file srep09222-s1.pdf]

Supplemental Information

Designed angiopoietin-1 variant, COMP-angiopoietin-1, rescues erectile function through healthy cavernous angiogenesis in a hypercholesterolemic mouse

Ji-Kan Ryu[1,2,*], Woo Jean Kim[1,*], Young Jun Koh[3], Shuguang Piao[1], Hai-Rong Jin[1], Sae-Won Lee[4,5], Min Ji Choi[1], Hwa-Yean Shin[1], Mi-Hye Kwon[1], Keehoon Jung[3], Gou Young Koh[3,†], and Jun-Kyu Suh[1,†]

[1]National Research Center for Sexual Medicine and Department of Urology, Inha University School of Medicine, Incheon 400-711, Republic of Korea
[2]Inha Research Institute for Medical Sciences, Inha University School of Medicine, Incheon 400-711, Republic of Korea
[3]National Research Laboratory of Vascular Biology and Stem Cells and Graduate School of Medical Science and Engineering, Korea Advanced Institute of Science and Technology (KAIST), Daejeon 305-701, Republic of Korea
[4]Department of Internal Medicine and Innovative Research Institute for Cell Therapy, Seoul National University Hospital, Seoul, Republic of Korea
[5]Biomedical Research Institute, Seoul National University Hospital, Seoul, Republic of Korea

[*]Ji-Kan Ryu and Woo Jean Kim contributed equally to this study.

[†]Correspondence to Jun-Kyu Suh, MD, PhD or Gou Young Koh, MD, PhD

Jun-Kyu Suh, MD, PhD
National Research Center for Sexual Medicine and Department of Urology
Inha University School of Medicine
7-206, 3rd ST, Shinheung-Dong, Jung-Gu, Incheon 400-711
Republic of Korea
Tel: 82-32-890-3441

Fax: 82-32-890-3097
E-mail: jksuh@inha.ac.kr

Gou Young Koh, MD, PhD
National Research Laboratory of Vascular Biology and Stem Cells and Graduate School of Medical Science and Engineering
Korea Advanced Institute of Science and Technology.
373-1, Guseong-dong, Daejeon, 305-701, Republic of Korea
Phone: 82-42-869-2638
Fax: 82-42-869-2610
E-mail:gykoh@kaist.ac.kr

Supplemental Methods

Cell culture experiments

Human umbilical vein endothelial cells (HUVECs) or mouse cavernous endothelial cells (MCECs) were prepared and maintained as previously described[1,2]. Primary cultured endothelial cells were grown on 0.3% gelatin-coated dishes in 2% serum medium (Cell Systems, Kirkland, WA, USA) or in complement medium 199 (M199, Gibco, Carlsbad, CA, USA) supplemented with 20% FBS, 3 ng/ml basic fibroblast growth factor, 5 U/ml heparin, and 1% penicillin/streptomycin. Cells were used between passages 2 and 4. HUVECs or MCECs were incubated with COMP-Ang1 protein (400 ng/ml/day) for three consecutive days then harvested 24 hours after treatment and used for western blot analysis of eNOS, phospho-eNOS, and cGMP expression. To investigate the signaling cascade involved in COMP-Ang1-mediated restoration of endothelial cell-cell junction proteins, MCECs were pretreated with chemical inhibitor of PI3K/Akt pathway (LY294002, Cell Signaling, Beverly, MA, USA) or MEK/ERK pathway (U0126, Calbiochem, Boston, MA, USA) 2 hours before COMP-Ang1 treatment. The cells were then harvested 12 hours after treatment of COMP-Ang1 and used for western blot analysis of occludin and claudin-5. MCECs were used for *in vitro* angiogenesis, cell proliferation, and cell permeability assay.

RNA interference

MCECs were grown to 70% confluence, and small interfering RNAs (siRNAs) against histone deacetylase 2 (HDAC2; 50 μM) were transfected into the cells using MetafectenePro (Biontex, San Diego, CA, USA). siRNA transfection was performed according to the manufacturer's instructions. HDAC2 siRNA and scrambled siRNA were purchased from Santa Cruz Biotechnology (Santa Cruz, CA, USA). The cells

were harvested 48 hours after treatment and used for RT-PCR and western blot analysis for HDAC2, occludin, and claudin-5.

Transient transfection

pcDNA3 inserted full-length HDAC2 gene was supplied from Dr. Kyu-Won Kim from Seoul National University (South Korea). Transient transfections were performed by using MetafectenePro (Biontex). All transfections were performed according to the manufacturer's instructions. Endothelial cells were transiently transfected with 3 μg HDAC2 expression vector after seeding on 60 mm culture dishes. To isolate RNA or proteins, medium was changed into M199 medium (Gibco) containing 1% FBS and endothelial cells were incubated for 24 hours. Endothelial cells were collected and filtered through a 0.22 μm pore size membrane (Millipore, San Francisco, CA, USA), and then concentrated four times using centrifugal filters (Millipore).

RT-PCR

Total RNA was isolated from the MCECs using Trizol reagent (Invitrogen, Eugene, OR, USA). PCR products were analyzed on 1.0% agarose gels and the gels were digitally imaged. The following mouse-specific primer pairs were used: HDAC2, 5'-CAT CCC ATG AAG CCT CAT AGA AT-3', 5'-CAC CAA TAT CCC TCA AGT CTC C-3', occludin, 5′-TCA GGC AGC CTC GTT ACA G-3′, 5′- CCT CCT CCA GCT CAT CAC A -3′, claudin-5, 5′-GGT CAC TGG GAA CTT CCT GA-3′, 5′- ATG CCA CTC ACT GCC TCT CT-3′. GAPDH was used as an internal control.

SDS-PAGE and immunoblotting

Proteins were extracted with lysis buffer (40 mM Tris PH 7.4, 10 mM EDTA, 120 mM NaCl, 0.1% NP-40, protease inhibitor cocktail, Roche Applied Science, Indianapolis, IN,

USA) and protein concentration was measured by Bradford assay. Total protein (30 μg/lane) was loaded, separated by SDS-PAGE and transferred onto nitrocellulose membrane (Amersham Biosciences, Sweden). The membranes were incubated for 1 hour with blocking solution (5% skim milk in 0.1% Tween-20 PBS) and then incubated for overnight at 4°C with antibodies to eNOS (Transduction Laboratories, Inc., Lexington, KY, USA), phospho-eNOS (Cell Signaling), HDAC2 (Santa Cruz Biotechnology), occludin (Zymed Laboratories, South San Francisco, CA, USA), and claudin-5 (Zymed Laboratories). Membrane was washed with 0.1% Tween-20 PBS and then incubated for 90 minutes with secondary antibody. The membrane was washed with 0.1% Tween-20 PBS and the signals were developed using ECL plus detection system (Amersham Bioscience). As an internal loading control, anti-β-actin antibody (Abcam, Cambridge, MA, USA) was used.

Measurement of cell proliferation

MTT assay was performed to identify cell viability. Briefly, siRNA-transfected MCECs were plated at a density of 5×10^3 cells per well in 96-well plates for 48 hours. After removing the medium, cells were treated with dimethylsulfoxide (DMSO). The final concentration of DMSO in the medium was ≤ 0.1% (v/v). After the cells were incubated for 48 hours, 20 μl MTT solutions (2 mg/ml) was added to each well for another 4 hours at 37°C. The formazan crystals that formed were dissolved in DMSO (200 μl/well) by constant shaking for 5 minutes. The plate was then read on a microplate reader at 540 nm. Three replicate wells were used for each analysis.

Wounding migration assay

MCECs were plated on 60 mm culture dishes at 95% confluence and were wounded with a razor blade 2 mm in width and marked at the injury line. After wounding, the

cultures were washed with serum-free medium and further incubated in M199 media with 1% serum and 1 mM thymidine. MCECs were allowed to migrate for 12 hours and were rinsed with serum-free medium, followed by fixing with absolute methanol and staining with Giemsa solution. Migration was quantitated with counting the number of cells that moved beyond the reference line.

Tube formation assay

50 μl of growth factor-reduced Matrigel (Collaborative Biomedical Products, Bedford, MA, USA) was dispensed into 96-well tissue culture plates at 4°C. After gelling at 37°C for at least 30 minutes, MCECs were seeded onto the gel at 2×10^4 cells/well in 200 μl of M199 medium. The assay was performed in a CO_2 incubator and the plates were incubated at 37°C for 24 hours. Images were obtained with a phase-contrast microscope and the numbers of tubes in each well of the plate were counted at a screen magnification of ×40.

Rhodamine B isothiocyanate-dextran permeability assay

Permeability across the endothelial cell monolayer was measured by using type I collagen-coated transwell units (6.5 mm diameter, 3.0 μm pore size polycarbonate filter; Costar, Cambridge, MA, USA). After MCECs become confluent, permeability was measured by adding 0.1 mg of rhodamine B isothiocyanate-labeled dextran (molecular weight, ~10,000) per milliliter to the upper chamber. After incubation for 15 minutes, 100 μl of sample from the lower compartment was diluted with 100 μl of PBS and measured for fluorescence at 635 nm when excited at 540 nm with a spectrophotometer (Tecan SpectraFluor; Tecan Durham, NC, USA).

Animals and treatments

Specific pathogen-free C57BL/6J and *Nos3*$^{-/-}$ (C57BL/6J genetic background) mice were purchased from Orient Bio (Gyeonggi, South Korea) and from The Jackson Laboratory (Bar Harbor, ME, USA) and bred in our pathogen-free animal facility. Two-month-old male mice were used in this study. The experiments were approved by the Institutional Animal Care and Use Subcommittee of Inha University. Control animals were fed a normal diet and experimental animals were fed a diet containing 4% cholesterol and 1% cholic acid (Feed Lab. Co., Gyeonggi, South Korea) for 3 months. The hypercholesterolemic mice were anesthetized with ketamine (100 mg/kg) and xylazine (5 mg/kg) intramuscularly and placed supine on a thermoregulated surgical table. The penis was exposed using sterile technique. A 30-gauge insulin syringe was used to administer a single injection of ad-LacZ (2×10^8 parts/20 µl) or ad-COMP-Ang1 (2×10^8 parts/20 µl) and repeated injections of BSA (days -3 and 0; 5.88 µg/20 µl,) or COMP-Ang1 recombinant protein (days -3 and 0; 5.88 µg/20 µl) into the mid portion of the corpus cavernosum as previously described[3]. The incision was closed with 6-O Vicryl (polyglactin 910) sutures. We evaluated erectile function (n=8 per group) and cavernous blood flow (n=4 per group) by cavernous nerve electrical stimulation 2 or 8 weeks after treatment. Cavernous specimens from a separate group of animals were used for histological and biochemical analyses (reverse transcription-polymerase chain reaction [RT-PCR], western blot, or cGMP quantification). Blood was extracted by direct cardiac puncture and serum total cholesterol level was determined using a commercially available kit (Boehringer Mannheim GmBH, Mannheim, Germany) and an automatic analyzer (HITACHI 7600, Hitachi Koki Co., Hitachinaka, Japan).

Immunohistochemistry and immunofluorescence

For immunohistochemistry, frozen tissue sections (20-µm thick) were incubated with an

antibody to FLAG M1 (Sigma-Aldrich, St. Louis, MO, USA), then treated with secondary antibodies and developed according to the instructions in the Histostain-SP kit (Zymed Laboratories). Subsequently, sections were stained with hematoxylin. For fluorescence microscopy, frozen tissue sections (20-μm thick) were incubated with antibodies to PECAM-1 (Chemicon International Inc., Temecula, CA, USA), claudin-5 (Zymed Laboratories), occludin (Zymed Laboratories), phosphohistone H3 (Upstate, Temecula, CA, USA), phospho-eNOS (Ser1177, Cell Signaling), HDAC2 (Santa Cruz Biotechnology), Ang1 (Abcam), Tie2 (Cell signaling), oxidized LDL (Abcam), or Cy3-conjugated antibody to smooth muscle α-actin (Sigma-Aldrich) at 4°C overnight. After several washes with PBS, the sections were incubated with tetramethyl rhodamine isothiocyanate-conjugated anti-hamster IgG antibody or fluorescein isothiocyanate-conjugated anti-rabbit antibody (Zymed Laboratories) for 2 hours at room temperature. Mounting medium containing 4,6-diamidino-2-phenylindole (DAPI; Vector Laboratories Inc., Burlingame, CA) was applied to the samples and nuclei were labeled when appropriate. Signals were visualized and digital images were captured using an Apotome microscope (Zeiss, Göttingen, Germany). The extent of PECAM1 staining was evaluated by calculating the labeled area (total labeled pixels) and the staining intensity (degree of labeling within the labeled area) using an image analyzer system (NIH Image J 1.34). Endothelial cell area was expressed as a percentage of the total cavernous area [(positive area/total cavernous area) × 100]. The numbers of phosphohistone H3-immunopositive endothelial cells were counted at a screen magnification of ×400 in eight different regions. Values were expressed per high-power field (HPF).

Time course of COMP-Ang1-specific mRNA expression in mouse corpus cavernosum

The expression of COMP-Ang1 mRNA in the corpus cavernosum was detected by RT-PCR 3, 7, 14, and 21 days after intracavernous injection of ad-COMP-Ang1. First-strand cDNA was synthesized using reverse transcriptase with oligo(dT) primers, and PCR was performed with a forward primer specific for the sequence encoding the FLAG tag (5'-GAC TAC AAA GAC GAT GAC GAC-3') and a reverse primer specific for an internal sequence in the human Ang1 portion of the transcript (5'-ACC TTC TTT AGT GCA AAG ATT GAC-3'). β-actin was used as an internal control. All signals were visualized and analyzed by densitometry.

SDS-PAGE and immunoblotting

The time course of COMP-Ang1 protein expression or persistence in the corpus cavernosum was detected by western blot at 1, 6, and 24 hours and 3, 7, 14, and 21 days after intracavernous injection of ad-COMP-Ang1 or COMP-Ang1 protein. We also evaluated the expression of eNOS and phospho-eNOS in control and hypercholesterolemic mouse penises 14 days after injection of the gene or protein (n=4). Equal amounts of protein were electrophoresed on 8% SDS-polyacrylamide gels, transferred to nitrocellulose membranes, and probed with antibodies to FLAG M1 (Sigma-Aldrich), eNOS (Transduction Laboratories, Inc.), phospho-eNOS (Ser1177, Cell Signaling), or β-actin (Abcam). Results were quantified by densitometry.

cGMP determination

At 2 and 8 weeks post intracavernous gene or protein injection (n=4 per each time point), the penile tissue was removed and rinsed with PBS, quick frozen in liquid nitrogen, and stored at -70°C until cGMP determination. The samples were then processed according to the instructions provided with the cGMP quantification kit (Cayman Chemical, Ann Arbor, MI, USA). Data are expressed as pmol/mg of wet

weight tissue.

References

1. Kim, I. *et al*. Angiopoietin-1 induces endothelial cell sprouting through the activation of focal adhesion kinase and plasmin secretion. *Circ. Res.* **86**, 952-959 (2000).
2. Yin, G.N. *et al*. Matrigel-based sprouting endothelial cell culture system from mouse corpus cavernosum is potentially useful for the study of endothelial and erectile dysfunction related to high-glucose exposure. *J. Sex. Med.* **9**, 1760-1772 (2012).
3. Lee, M. *et al*. Water-soluble lipopolymer as a gene carrier to corpus cavernosum. *Int. J. Impot. Res.* **17**, 326-334 (2005).

Supplemental Figure Legends

Supplemental Figure 1. Adenoviral COMP-Ang1 gene or COMP-Ang1 protein transfer induces cavernous Tie2 expression. Immunohistochemical staining of cavernous tissue using antibodies to Ang1 (red), Tie2 (red), and PECAM-1 (CD31; green) in age-matched control or hypercholesterolemic mice 2 weeks after intracavernous injection of ad-LacZ (L, 2×10^8 parts/20 μl), BSA (B, 5.88 μg/20 μl), ad-COMP-Ang1 (CA, 2×10^8 parts/20 μl), COMP-Ang1 protein (CP, 5.88 μg/20 μl), or cholesterol diet only (CH). Scale bar = 200 μm.

Supplemental Figure 2. Optimal dosage for inducing maximal erectile responses. Intracavernous pressure (ICP) responses during stimulation of the cavernous nerve for age-matched control (C) or hypercholesterolemic mice 2 weeks after a single intracavernous injection of ad-LacZ (L, 2×10^8 parts/20 μl), repeated injections of BSA (B, days -3 and 0; 5.88 μg/20 μl), a single injection of ad-COMP-Ang1 (CA, 2×10^7, 1×10^8, 2×10^8, 1×10^9, 2×10^9, or 1×10^{10} parts/20 μl), a single injection of COMP-Ang1 protein (CP, 5.88 μg/20 μl), repeated injections of COMP-Ang1 protein (CP, days -3 and 0; 5.88 μg/20 μl), or cholesterol diet only (CH). Ratios of mean maximal ICP and total ICP (area under the curve) to mean systolic blood pressure (MSBP) were calculated for each group. Each bar depicts the mean ± SEM for n=6 or 8 animals per group. One-way ANOVA was used for statistical analysis. **(a)** Ratio of maximal ICP to MSBP. $^*p < 0.01$ vs C, CA (2×10^8 parts/20 μl), and CP (repeated injections) groups, †$p < 0.01$ vs C group, ‡$p < 0.05$ vs CA (2×10^8 parts/20 μl) and CP (repeated injections) groups, $^{**}p < 0.05$ vs C group, #$p < 0.01$ vs CH, B, and CA (1×10^{10} parts/20 μl) groups, ##$p < 0.05$ vs L group. **(b)** Ratio of total ICP (area under the curve) to MSBP. $^*p < 0.01$ vs C, CA (2×10^8 parts/20 μl), and CP (repeated injections) groups, †$p < 0.01$ vs C group, ‡p

< 0.05 vs CA (2 × 10^9 parts/20 μl) and CP (single injection) groups, #p < 0.01 vs CH and B groups, ##p < 0.05 vs CH and B groups.

Supplemental Figure 3. Adenoviral COMP-Ang1 gene or COMP-Ang1 protein transfer increases cavernous endothelial content through enhanced endothelial cell proliferation. (a) Immunohistochemical staining of cavernous tissue using antibodies to PECAM-1 (CD31; red) and phosphohistone H3 (PH3; green) in age-matched control or hypercholesterolemic mice 2 weeks after intracavernous injection of each gene or protein. Scale bar = 50 μm. **(b)** Immunohistochemical staining of cavernous tissue performed with antibodies to PECAM-1 and PH3 1, 3, 6, and 12 hours after intracavernous injection of COMP-Ang1 protein (5.88 μg/20 μl). Scale bar = 50 μm. **(c,d)** Number of PH3-immunopositive endothelial cells per high-power field (HPF, screen magnification ×400). Each bar depicts the mean ± SEM for n=6 (**c**) or n=4 (**d**) animals per group. One-way ANOVA was used for statistical analysis. **(c)** Two weeks after treatment with each gene or protein. #p < 0.01 vs C, CH, L, B, and CP groups. **(d)** One hour, 3, 6, and 12 hours after treatment of COMP-Ang1 protein. #p < 0.01 vs C, 1-h, and 12-h groups. ##p < 0.01 vs 3-h group.

Supplemental Figure 4. Adenoviral COMP-Ang1 gene transfer decreases cavernous HDAC2 expression. (a) Immunohistochemical staining of cavernous tissue using antibodies to smooth muscle α-actin (red) and HDAC2 (green) in age-matched control or hypercholesterolemic mice 2 weeks after intracavernous injection of ad-LacZ (2 × 10^8 parts/20 μl), ad-COMP-Ang1 (2 × 10^8 parts/20 μl), or cholesterol diet only. Scale bar = 200 μm. **(b,c)** Immunohistochemical staining of cavernous tissue performed with antibodies to PECAM-1 (red), smooth muscle α-actin (red), or HDAC2 (green) in hypercholesterolemic mice. Note significant expression of HDAC2 in

endothelium of the cavernous sinusoid and cavernous artery (yellow color on merged image). Scale bar = 50 μm. DAPI, 4,6-diamidino-2-phenylindole (blue); SMCs, smooth muscle cells.

Supplemental Figure 5. siRNA mediated silencing of HDAC2 did not affect on cell viability, migration, and tube formation in MCECs. (a) Cytotoxicity of HDAC2 siRNA to MCECs. Scrambled or HDAC2 siRNA was added to the cells and incubated for 48 hours at 37°C. After the incubation, cell viability was measured by MTT assay. Each bar depicts the mean ± SEM of four independent experiments. Mann-Whitney *U*-test was used for statistical analysis. **(b)** Migration assay. Scrambled or HDAC2 siRNA was added to the MCECs and cells were wounded with a razor blade 2 mm in width. Then, the cells were allowed to migrate for 12 hours and migration was quantitated with counting the number of cells that moved beyond the reference line. Each bar depicts the mean ± SEM of four independent experiments. Mann-Whitney *U*-test was used for statistical analysis. **(c)** Tube formation assay. Phase-contrast microscopy of MCECs (screen magnification ×40). After serum starvation for 24 hours, MCECs were transfected with scramble siRNA or siRNA specific to HDAC2 for 48 hours. Then, the tube formation assay on Matrigel was performed in 96-well dishes.

Supplemental Figure 6. Biodistribution of ad-COMP-Ang1. A representative gel showing the distribution of COMP-Ang1 transcripts in corpus cavernous tissue and other major organs 3, 7, 14, and 21 days after intracavernous administration of ad-COMP-Ang1 (2×10^{8} parts/20 μl). PCR was performed with primers specific for COMP-Ang1. Untreated HUVECs (**HUVEC [-]**) and HUVECs transfected with ad-COMP-Ang1 (**HUVEC [+]**) were used as negative and positive controls, respectively. Results were similar in three independent experiments. M, marker.

Supplemental Table

Table 1 Physiologic and metabolic variables

		Cholesterol diet				
	Control	Diet only	Ad-LacZ	BSA	Ad-COMP-Ang1	COMP-Ang1 protein
Body weight (g)						
2 weeks after Tx.	32.5 ± 0.8	25.9 ± 0.4*	23.8 ± 1.2*	26.4 ± 1.2*	24.9 ± 0.9*	25.0 ± 1.0*
8 weeks after Tx.	32.8 ± 0.8	24.6 ± 1.0*	26.9 ± 0.7*	25.1 ± 0.9*	27.6 ± 0.4*	25.5 ± 1.0*
Blood pressure (mm Hg)						
2 weeks after Tx.						
SBP	96.4 ± 1.4	99.5 ± 2.3	99.9 ± 2.8	101.3 ± 3.2	96.3 ± 5.0	101.5 ± 3.5
MBP	77.1 ± 1.2	73.6 ± 2.3	78.9 ± 2.1	79.6 ± 2.8	72.7 ± 3.5	77.5 ± 3.5
DBP	64.5 ± 1.7	60.2 ± 2.6	68.5 ± 2.5	68.8 ± 2.9	61.5 ± 3.2	65.4 ± 4.3
8 weeks after Tx.						
SBP	97.2 ± 1.1	101.6 ± 2.2	95.8 ± 2.9	98.1 ± 2.5	96.1 ± 3.3	101.6 ± 2.9
MBP	75.1 ± 1.2	77.1 ± 2.5	76.5 ± 2.9	76.4 ± 2.5	71.9 ± 2.3	75.7 ± 3.2
DBP	63.9 ± 1.7	64.0 ± 2.9	66.7 ± 3.1	65.6 ± 2.7	60.3 ± 2.5	62.6 ± 3.7
Total cholesterol (mg/dl)						
2 weeks after Tx.	100.5 ± 3.4	180.3 ± 6.8†	192.9 ± 12.6*	184.6 ± 7.3†	192.2 ± 23.1*	190.8 ± 18.2*
8 weeks after Tx.	80.7 ± 3.5	228.5 ± 24.9*	200.1 ± 21.9*	190.8 ± 16.4*	208.3 ± 10.1*	206.3 ± 17.3*

Values are the mean ± SEM for n=8 animals per group. One-way ANOVA was used for statistical analysis. *p < 0.01, †p < 0.05 vs control group. Ang1, angiopoietin-1; SBP, systolic blood pressure; MBP, mean blood pressure; DBP, diastolic blood pressure.

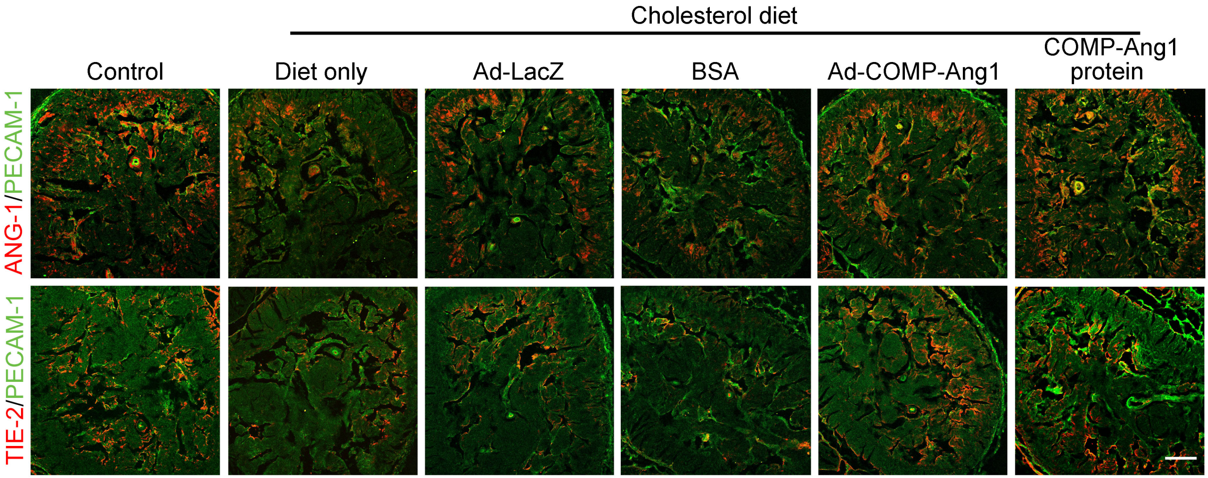

Supplemental Figure 1

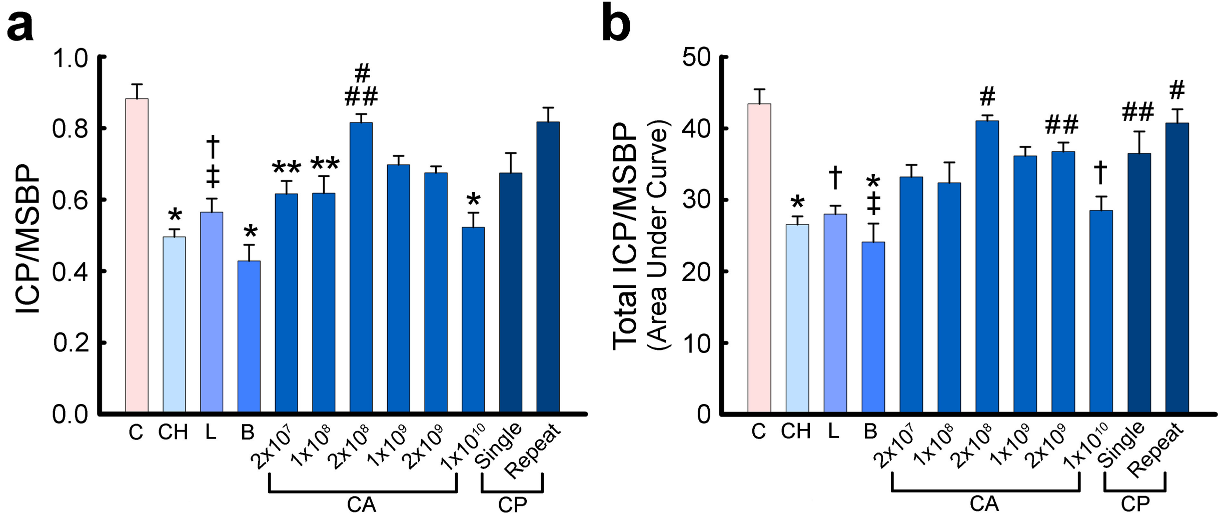

Supplemental Figure 2

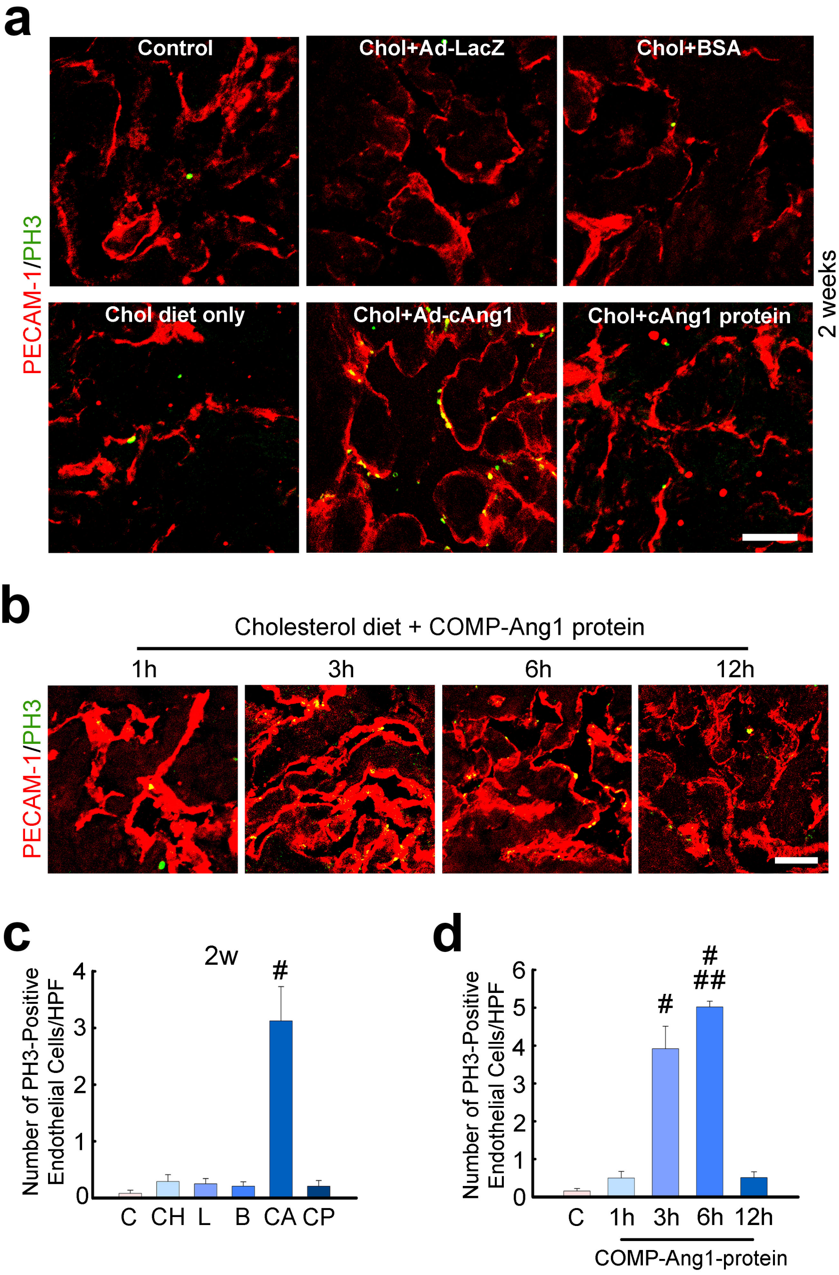

Supplemental Figure 3

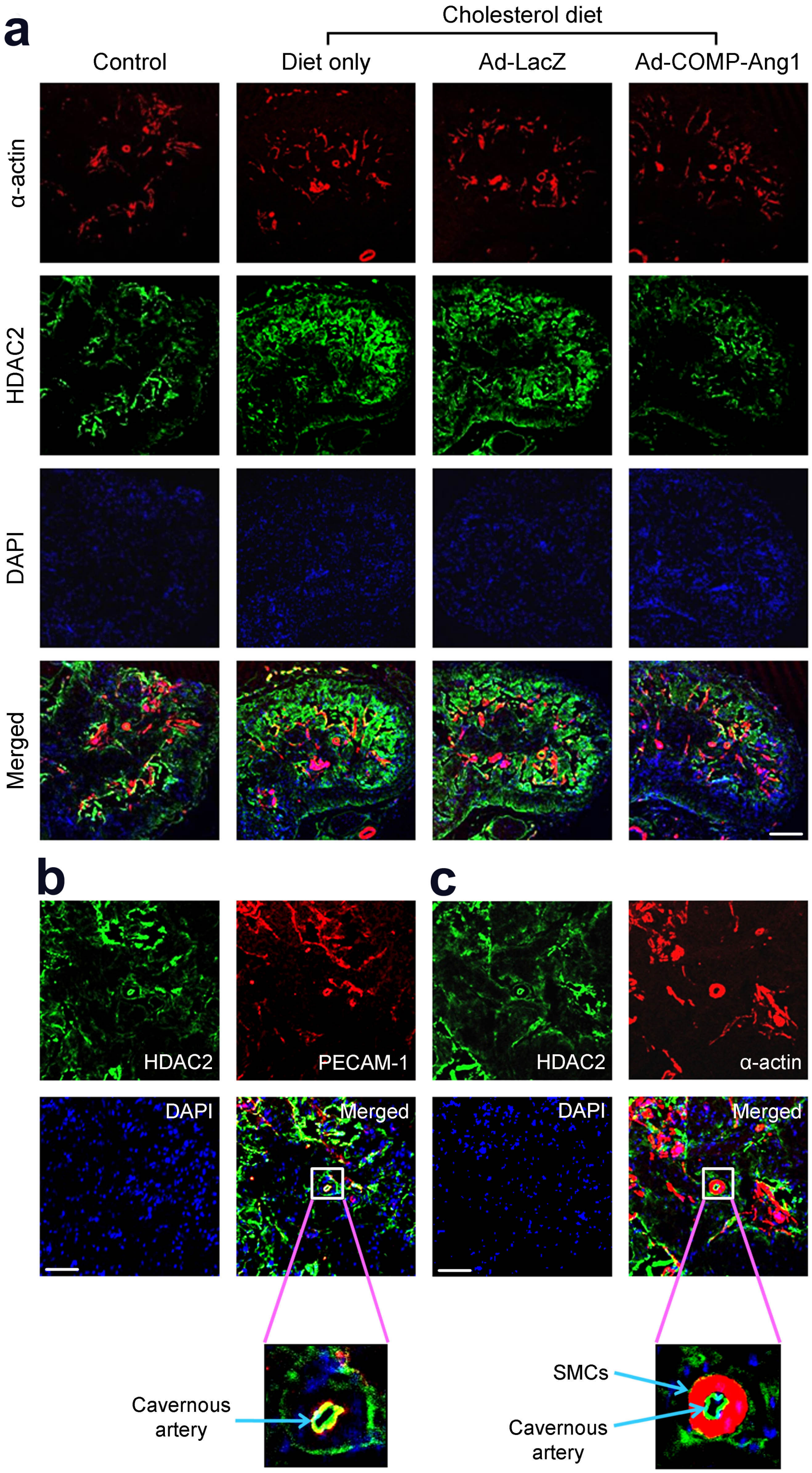

Supplemental Figure 4

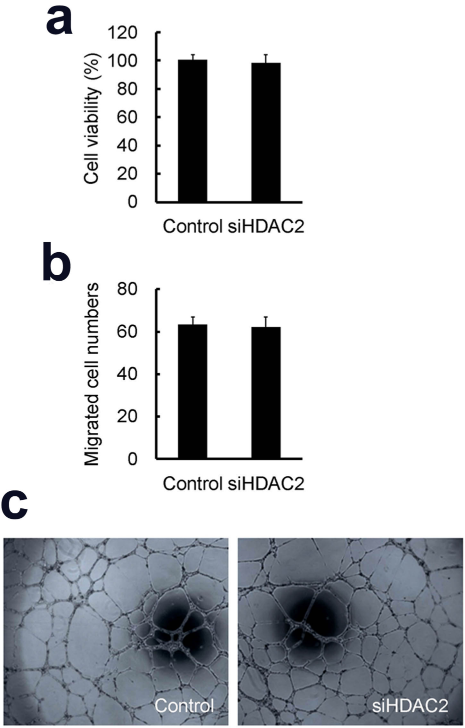

Supplemental Figure 5

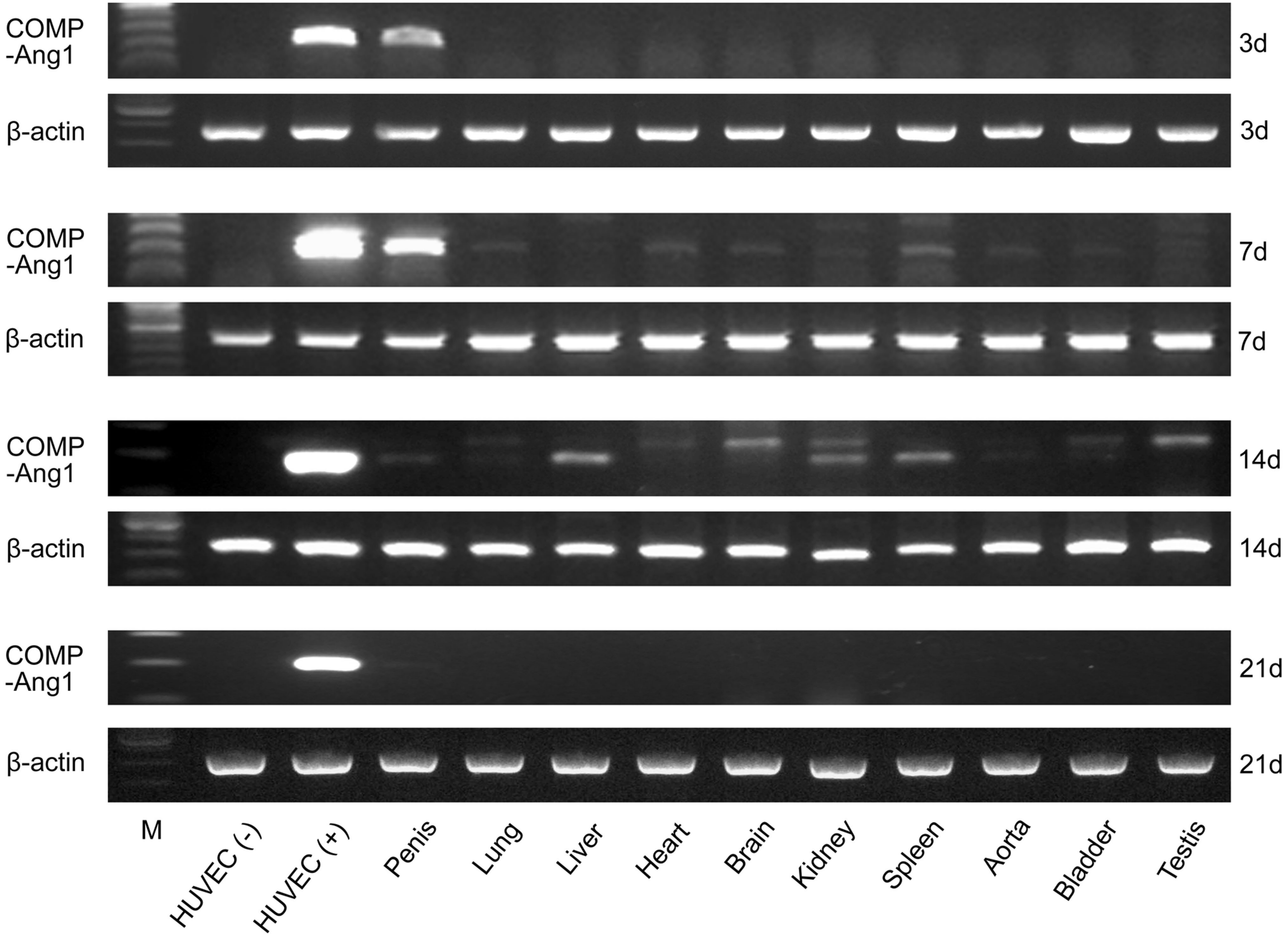

Supplemental Figure 6
